# Medicine

**Published:** 1913-12

**Authors:** J. M. Fortescue-Brickdale


### Progress of the Medical Sciences.

### MEDICINE.

**The Pancreatic Secretion.**—An interesting question has been raised by Schlagintweit and Stepp[1] as to the effect of achylia gastrica on the pancreatic secretion. As is well known, the secretion of pancreatic juice is provoked by a chemical reflex, namely by the formation of secretin, and its conduction to the pancreas by way of the blood stream. But as secretin is evolved from the duodenal mucosa by the action of the acid gastric juice, the authors imagined that possibly where the latter was inactive or absent some deficiency in pancreatic secretion might secondarily occur. They therefore devised two series of experiments to test this view. First, they obtained specimens of gastric juice from normal persons and from those suffering from carcinoma of the stomach and from achylia. These they mixed with extract from the duodenal mucosa, and injected intravenously into dogs. They found that normal and most carcinomatous persons gave a juice which produced secretin, and caused a flow of pancreatic secretion, but that in achylia the results were negative. They then successfully operated on a healthy bitch, in order to produce a duodenal and pancreatic fistula. The animal remained in normal health after the operation. Into the duodenal fistula they injected gastric juice from normal persons, from those with superacidity and subacidity, from those with undoubted carcinoma, and also salt solutions of various strengths. The injections were always made when the animal's stomach was empty, slowly and under low pressure, and due note was taken of any instances when the animal's own gastric juice escaped into the duodenum. As a result of many observations, they found that the injection of anacid gastric juice either from cases of carcinoma or from cases of achylia only produced a minimal pancreatic secretion.

This result is opposed to the commonly accepted view that when gastric digestion is eliminated the pancreas takes on a compensatory over-action. The authors discuss possible criticisms of their findings ; it may be urged that in diseased persons the pancreas is activated (*i.e.* secretin is formed) apart from the action of the gastric juice, and that it is impossible to apply the results obtained from dogs to the human subject until a condition analogous to achylia gastrica has been proved to occur in these animals. Still, they are of opinion that considerable importance attaches to their experiments, especially

[1] *München. med. Wchnschr.*, 1913, lx. 1865.

as there are no systematic observations of the trypsin secretion in cases of achylia. Von Noorden has indeed shown that the absorption of food is not disturbed in achylia, but on the other hand cases have been observed where achylia gastrica was accompanied by absence of pancreatic secretion. They are inclined to think that there may be other substances, such as oils, which excite pancreatic secretion by a nervous reflex, and that soaps formed in the intestine may produce duodenal secretin. For these they quote some experimental evidence, and also mention some recent researches which show that a rhythmic and automatic flow of pancreatic juice may occur. They express no opinion as to the question of psychic influence on pancreatic secretion, but conclude that at any rate it is the result of several different causative factors, and when the less important ones are in abeyance the absence of acid gastric juice leads to severe pancreatic insufficiency (pancreatic achylia).

The effect of the absence of bile from the intestine has been studied in a child aged ten weeks, suffering from congenital absence of the bile-ducts, by Koplik and Crohn.[1] The pancreatic duct was normal, and the only deficiency concerned the splitting up and absorption of fats. Nitrogen metabolism was unaffected, and the pancreatic secretion active. Höflich[2] has shown in twelve cases that severe disease of the gall-bladder and cholecystectomy fails to influence the secretion of gastric juice, so that presumably the pancreatic secretion is also unaffected.

**Gastric and Duodenal Ulceration.**—Many theories have been evolved as to the causation of gastric ulceration, two of the best known being that which attributes the condition to embolic necrosis, and that first put forth as the result of experimental work by Bolton, which supposes that cytotoxins or more or less specific cellular poisons acting in an acid medium are the cause both of the ulcer and its chronicity. The bacterial origin of these ulcers has also, however, been somewhat widely held, and from this point of view Steinharter[3] has carried out some interesting experiments. He prepared broth cultures of bacillus coli, and caused them to clump by the addition of an acid, agglutinating reagent. The culture was centrifugalised, and an emulsion of clumped bacilli obtained and injected into the ear vein of a hare or rabbit. In five animals ulceration or some evidence of destruction of the epithelial cells was found. In one previously immunised animal hemorrhages but no ulceration occurred; and two controls, one of which was not injected, and the other only with an emulsion containing the agglutinating reagent, were normal. The author supposes that

[1] *Am. J. Dis. of Childr.*, 1913, v. 36.

[2] Abstract in *Schmidt's Jahrbuch*, 1913, cccxviii. 242.

[3] *Boston M. & S. J.*, 1913, clxix. 81.

similar processes may occur in the human subject, and proposes further investigations as to the exact mode in which the lesion is produced. Thus it may be embolic or toxic or both ; it may be selective or general ; other organisms may be able to produce similar effects. No statement is made in the present communication as to the condition of the other organs of the experimental animals, and the author's further investigations will be awaited with interest.

Flesch[1] in an analysis of the post-mortem appearances of eleven cases of duodenal and gastric ulcer in infants under six months of age, lays stress on the importance of two ætiological factors in these cases. Firstly, the absence of antipepsin in marasmic infants, and secondly, the situation above the entrance of the pancreatic and bile-ducts where these ulcers are usually found, and where the mucosa is not protected by alkaline secretions.

The **diagnosis** of gastric and duodenal ulceration by means of the X-rays is the subject of a paper by F. W. White and A. W. George.[2] They point out that in all cases *two* series of plates should be taken, in order to obtain confirmatory evidence of the appearances. With regard to *gastric ulcer*, two important changes may be noted. The first is local or reflex pyloric spasm ; the former intermittent, and best seen when the stomach is empty. The reflex pyloric spasm lasts for a very variable time, and causes considerable delay in the evacuation of the stomach contents into the duodenum. Generally, they consider that a considerable residue after six hours, associated with superacidity and capable of no anatomical or other explanation, is suggestive of gastric ulceration, though in half the cases of ulcer the stomach is empty in six hours. The second important change is in the shape of the stomach. This may be due to cicatricial contraction, which produces the well-known hour-glass stomach. The contracting ring is then constant, immobile, and nearer the lower end of the organ. The stomach may be drawn over towards the left. External adhesions may cause great variations in the shape of the stomach according to their position and origin. Those between the stomach and gall-bladder may pull the former over to the right. The causes of the adhesions cannot usually be determined by X-ray examination, though they are usually due to old ulceration or disease of the gall-bladder. The authors also draw attention to the pouch of bismuth sometimes seen in perforating ulcers. The air bubble at the apex of the pouch they have not found at all constant ; the lateral view is recommended for discovering perforating ulcers on the posterior surface.

With regard to **duodenal ulcers,** the authors insist on the

[1] *Jahrb. f. Kinderheilk.*, 1912, lxxxi. 542.

[2] *Boston M. & S. J.*, 1913, clxix. 157.

importance of examining the patient from various points of view, lying down, standing up, or lying on the right side. The first part of the duodenum, the "bulbus duodeni" or "cap," which is important from the frequency with which lesions occur in it, should be seen in normal persons in one or other of these positions. Any marked or constant irregularity in its shape is indicative of some lesion, though shallow erosions may be present without influencing the X-ray picture. Both here and in other parts of the duodenum the abnormal shadows are produced by the results of ulceration or adhesions, and not by their simple presence alone. The authors consider stenosis of the duodeno-jejunal angle not very rare, and productive of great dilatation, but not frequently of ulceration. The vexed question of the influence of duodenal lesions on the mobility of the stomach is discussed, and the general conclusion reached that *per se* an ulcer in the duodenum causes the stomach to empty itself rapidly, but that when, as in cases of longer standing, some mechanical obstruction by adhesions or cicatricial contractions is present, this tendency is counteracted in greater or less degree, so that the time of evacuation may be normal or extended.

**The Action of Purgatives.**—Meyer-Betz and Gebhardt[1] have investigated the action of several purgatives by means of the bismuth meal in practically normal young men. Senna was found to have a very constant action; the small intestine was uninfluenced, and the cæcum was filled in the normal time, but emptied itself more rapidly. The main effect was on the large intestine, and especially on the transverse colon, well marked and rapid segmentation being observed, which appeared to progress in a regular and orderly way. They did not note any suppression of normal antiperistalsis. Extract of aloes did not give such constant results, but in like manner failed to affect the stomach and small intestine. Small doses produced an appreciable effect on the large intestine; big doses, while somewhat similar in their effect to senna, also produced deep segmentations, closely resembling in appearance a spastic constipation. The small fæcal masses were eventually collected together in the splenic flexure, and then passed on in small balls to the sigmoid. Thus large doses of aloes are apt to cause delay in the passage of the intestinal contents. Castor oil delayed the evacuation of the stomach, but hastened peristalsis in both small and large intestines, and caused abundant gas formation. The contents were moved forward by large spasmodic movements. Jalap produced no marked effect on the stomach, but caused excessive secretion and fluidity of the intestinal contents both in the small and large intestine. The cæcum was rapidly emptied, and defæcation began as soon as the rectum was filled.

[1] *München. med. Wchnschr.*, 1912, lix. 1793.

Salts when given with a meal did not hasten the emptying of the stomach, but when given later had a marked effect on the further evacuation of the viscus. The action on the small intestine was similar to that of jalap, but in the large intestine the fluid mass, mixed with gas, remained passive for some time, till a sudden explosive movement hurried it rapidly on, often, however, leaving the more solid portions behind. Magnesium sulphate given in equal doses acted more violently than Carlsbad salts. Calomel acted on both small and large intestine, without increasing the intestinal secretions.

A similar investigation, undertaken by Lebon and Aubourg[1] with reference to phenolphthalein, showed that small doses acted in the same way as scammony and jalap, but did not show evidence of increased secretion. Doses of scammony from $\frac{1}{2}$ to 1 gram ($7\frac{1}{2}$ to 15 grains), given in a cachet, showed little effect on the small intestine so far as increased peristalsis was concerned, though the fluidity of the intestinal contents was considerably increased with the larger dose.

J. M. FORTESCUE-BRICKDALE.

